# Cross Sectional Study Evaluating Routine Contact Investigation in Addis Ababa, Ethiopia: A Missed Opportunity to Prevent Tuberculosis in Children

**DOI:** 10.1371/journal.pone.0129135

**Published:** 2015-06-17

**Authors:** Dawit Assefa, Eveline Klinkenberg, Genet Yosef

**Affiliations:** 1 Monitoring and Evaluation unit, KNCV Tuberculosis Foundation TB CARE I country office, Addis Ababa, Ethiopia; 2 Research unit, KNCV Tuberculosis Foundation, Hague, Netherlands; 3 Department of Global Health, Amsterdam Institute for Global Health and Development, Academic Medical Center, Amsterdam, Netherlands; 4 TB/HIV control department, Addis Ababa City Administration Health Bureau, Federal Ministry of Health, Addis Ababa, Ethiopia; UCL Institute of Child Health, University College London, UNITED KINGDOM

## Abstract

The 2013 global roadmap for childhood tuberculosis calls for countries to implement contact screening and provide preventive therapy to children younger than 5 years. Therefore, this study designed to evaluate the implementation status of child contact screening and management in the health facilities of Addis Ababa, Ethiopia. Smear positive TB patients living with children attending daily observed treatment at the TB clinic and health care workers providing service were approached to address the study objective. Structured questionnaires were administered to smear positive index cases living with children whether they were requested to bring children age five year and below for TB screening and to health care providers in HIV, TB and child health clinics to assess their knowledge and practice on contact screening and management. Double data entry and analysis was done using EpiData software 3.1. In 27 health centres, 688 smear-positive index tuberculosis patients were approached of whom 203 (29.5%) reported to have children five years and below in their household. A total of 48 (23.6%) index cases had been requested by the health care workers to bring their children for tuberculosis screening and 45 (93.8%) had complied with this request. Of 230 children living with index smear positive tuberculosis patient, 152 (66.1%) were not screened for tuberculosis, 78 (33.9%) children screened, 2 had tuberculosis, 76 screened negative of which 3 (3.8%) received preventive treatment. None of the health care workers indicated to routinely record and report on child contact management. Household child contact screening and preventive intervention was sub-optimal in Addis Ababa. An important opportunity lost to prevent tuberculosis in young children. Training of health care workers, availing simple symptom based screening tool, and proper documentation could improve implementation.

## Introduction

Globally, over half a million children fall ill with tuberculosis (TB) each year and struggle with treatment that is not child friendly [1]. The long standing neglect of childhood TB has recently been addressed by the renewed commitment of the global TB community to address the burden of TB in children. The Roadmap for childhood TB was launched in 2013. The overall global TB control goal of reaching zero TB deaths in children was also adopted. Key actions and investments that are urgently needed to tackle childhood TB are outlined, for example, “Do not miss critical opportunities for intervention such as use of strategies for intensified case-finding, contact tracing and preventive treatment” [1].

Ethiopia is one of the 22 high TB burden countries. Of the 131,677 TB cases of all forms notified in 2013, 16% (21,317) were among children below 15 years of age [2]. In countries with high burden of TB, around 10–20% of all TB cases are expected to occur in children [1]. Though there are no a direct incidence estimates for TB in children, a recent modelling study to estimate the burden of childhood TB in the 22 high burden countries has shown that the incidence of pediatric TB is higher than the number of notifications, particularly among young children [3].

Children are often in close contact with adults, and any child that lives in a setting where there are persons with infectious TB is at risk of contracting TB. The likelihood of infection following exposure is greatest when it is close contact (e.g. household) and if that person has sputum smear-positive disease [4]. For instance, among household contacts, the risk for secondary tuberculosis was greater in young children compared to adults (10% versus 1.9% respectively) [5]. Therefore, in high TB burden setting contact screening and preventive treatment in children less than 5 years of age need to be prioritized and implemented. The national TB and TB/HIV program guidelines recommend that all children who are close contacts of pulmonary TB patients should be screened for TB and managed accordingly.

Primary health care is organized in Ethiopia as the lowest level of care where five health posts (one health post per 5000 population) linked with one health center (one health center per 25,000 population) and with one next higher level of care which is a district hospital. There is a national TB/HIV training manual for Health Care Workers (HCWs) at all levels of care and this manual specifically address basic childhood TB including contact screening and management. Training of HCWs is an ongoing capacity building activity through the national TB program in collaboration with partners supporting the national TB program. HCWs at the primary health care level are considered qualified and expected to perform TB screening including children who have a contact history with a smear positive TB patient. Basic laboratory services like microscopy for acid fast bacteria (AFB) are available at health center level; however, chest X-ray (CXR) is available at district or higher level hospitals. According to the national guideline, a household child contact that was screened negative for active TB disease should be protected against TB with a six-month course of Isoniazid preventive treatment (IPT), for which 100mg INH doses are procured by the national TB program. However, uninterrupted supply of INH in general and 100mg dose INH in particular has been commonly reported as a problem by HCWs. In order to assist HCWs, the national guideline outlines a clinical algorithm of symptom based TB screening including TST and CXR [6, 7]. However, TST is not available in Ethiopia and CXR services are only found at higher level of care (hospitals). Job aids for HCWs like the TB/HIV screening algorithm, weight-band dosing of anti-TB drugs, etc are found in the national TB/HIV guideline but were not usually printed and made available for quick references at HF level.

Though the national guideline recommends child contact screening and management, there is no routine information available on this in the national TB program. Therefore, it is difficult to know the status of the implementation of household child contact screening and management. With the renewed attention for childhood TB globally and for intensified prevention and control efforts of childhood TB in the country, it is important to investigate the status and barriers related to the implementation of child contact screening in the country and make practical recommendation to overcome observed challenges.

## Materials and Methods

The study was conducted in the primary health care setting of Addis Ababa, the capital of Ethiopia. Addis Ababa was selected because it is an urban setting where access to service is assumed to be feasible to implement contact screening and management as per the national guidelines recommendation. Administratively the town is divided into 10 districts. At the time of the study 54 public health centers were providing Directly Observed Treatment (DOTS) service.

A cross sectional study design was employed to interview smear positive PTB index patients and HCWs from Sept 16 –Dec 13, 2013, using structured questionnaires (S1 and S2 Files). Sample size estimation to interview smear positive PTB patients was done using as key outcome indicator, the proportion of index cases that bring their household child contact for TB screening. Based on earlier studies in Malawi this was assumed to be around 10% [8, 9]. Using this with the formula to estimate a single proportion with an absolute precision of 5%, 95% confidence and 80% power a crude sample size of 132 was obtained. Doubling for clustering and 85% participation rate resulted in a target sample size of 304 index cases with children in their household.

Taking into consideration of an average notification rate of 8 smear positive PTB patients per HF per month and with an assumption of 45% of smear positive PTB patients would be living in same household with children ≤ 5 yr of age, from the 27 selected high volume HFs, we estimated to approach 648 smear positive PTB patients in three months time to reach our targeted sample size of 304 index cases.

To ensure geographic coverage, health facilities from all 10 sub-cities of Addis Ababa were included. Health facilities were selected based on the criteria of higher case notification of ‘all forms’ of TB ≥ 50 per quarter and duration of DOTS service (≥ 2 yrs), a longer duration of service related with higher patient load and possibility of training of HCWs by the health office. These criteria resulted in selection of 27 out of the 54 health facilities in Addis Ababa.

In the selected facilities, all smear positive TB patients who were on treatment for at least two weeks until their end of treatment at the time of data collection were identified in the health facility TB registers. It was assumed that contact screening should have happened within the first two weeks of initiating treatment for the index cases. All those identified were asked whether they have household members of children age five years or below during their daily DOTS visit and those who confirmed were approached to take part in the study.

Our approach to select HF for interviewing HCWs working in TB, HIV and child health clinics was since there are 10 sub-cities in Addis Ababa, from the 27 selected HFs for patient interview, we have picked 2 HFs per sub-city so that we would have equal representation of each sub-city and that make a total of 20 health facilities, where we have been able to interview a total of 60 HCWs in all sub-cities.

Key variables collected in this study were, the number of children 0–5 years of age who are household contacts of smear positive TB patients, the proportion of those children screened for TB and the proportion who received IPT. Patient and health system related factors on the implementation of contact investigation and management were also assessed in order to make appropriate recommendation to the Ministry of Health. Both open and closed types of questions were used to interview patient and care providers. Closed type of questions was used to gather information on demographic, number contact screened and received preventive treatment. Open ended questions were used to assess opinions and practice.

Data collection was conducted from September 16 to December 13, 2013. Data collection was done by trained nurses who were new graduate and were unemployed at the time of data collection. They were weekly supervised by the principal investigator and community TB officer. Collected data forms were checked for completeness. Double data entry, data validation and analysis were done using EpiData 3.1 software.

### Ethics Statement

The study protocol was submitted and approved by the ethics committee of Addis Ababa regional health bureau. It was considered that verbal consent appropriate taking in to account of illiterate participants. Information statement with consent form in the study protocol was submitted and the procedure to obtain oral consent was approved by the ethics committee. Obtaining consent was by the research interviewers using the prepared information sheet as a guide for verbal explanation and they provided pertinent information for participants on the overall purpose, risk and benefit of participation in the study and reassured that participation is voluntary and non-participation would not affect the care or service obtained from the health facility. Consent obtained was signed and dated by the interviewer on the consent form. Participants were all adults and no personal identifiers were recorded on the questionnaires.

## Results

In the selected 27 health centers a total of 688 smear positive index TB patients were approached. Of them 203 (29.5%) were eligible to participate as they had children of 5 years and below in their household (Table 1). All 203 approached index cases enrolled in the study. Over three quarter of the index TB patients, 160 (78.8%) had completed elementary and secondary school level. Their mean age was 32.7 years (standard deviation (SD) 15.5 years) and 101 (50%) had a monthly income of less than 25 USD. Eighty-one percent of the index cases were new smear positive cases (Table 2).

**Table 1:** Distribution of child contacts per household index cases.

Number of children ≤ 5 years per household	Number of households (N, %)	Total number of children (N = 230)
0	485, 70.5%	0
1	177, 25.7%	177
2	25, 3.6%	50
3	1, 0.15%	3

**Table 2:** Type of TB of the 203 index cases who have child contacts in their households.

Type PTB	Female	Male	N (%)
New smear positive PTB	83	82	165 (81.3%)
Retreatment PTB	14	18	32 (15.8%)
MDR-TB	2	4	6 (3%)
Total	99 (48.8%)	104 (51.2%)	203 (100%)

**Fig 1:**
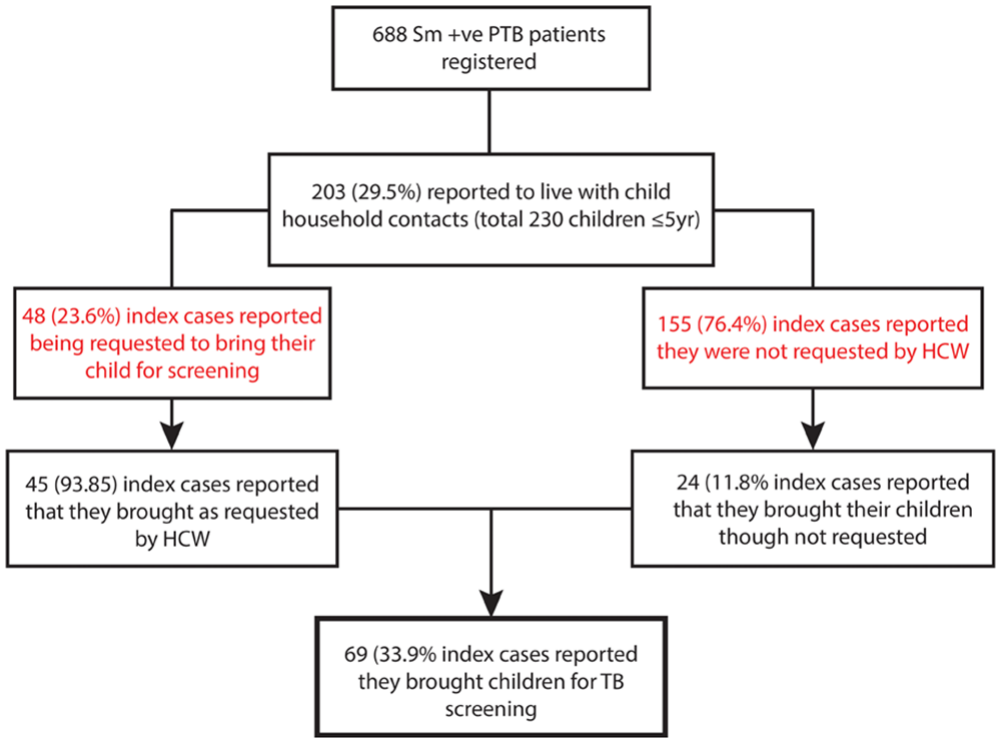
Flow diagram outlining the proportion of index PTB patients reported to bring their household child contact for tuberculosis screening.

From the 203 index cases, 48 (23.6%) reported that a HCW requested them to bring their child for TB screening, and 45 (93.8%) of them have brought their child contacts. In addition, despite not being requested, 24 index cases reported that they have brought their child contact for TB screening (Fig 1). Overall, of the 203 index cases, 69 (33.9%) index cases reported they have brought their child for TB screening (Table 3). A total of 134 index cases did not bring their children for TB screening. The main reported reasons for this were, 53 (39.6%) index cases said “I don’t know that the child should be screened for TB” while 52 (38.8%) said “the child is healthy and I don’t think it was necessary”. Five participants (3.9%) responded that “they had consulted HCWs who told them it was not necessary to screen” and 24 (21.6%) said “I don’t have time, money, etc”. Index cases were also requested on the source of information about child contact TB screening. Sixty percent of them indicated that health care workers at the TB clinic were the main source of information (Table 4).

**Table 3:** Smear positive PTB index patients and reported household child contacts with TB screening outcome.

Sm +ve PTB index cases		Children 1-5yrs household contacts		Outcome of children TB screened	
Index cases reported to bring their child contacts	69 (33.9%)	Children screened for TB	78 (33.9%)	Children screened negative	76 (97.4%)
Index cases reported they didn’t bring their child contacts	134 (66%)	Children not screened for TB	152 (66%)	Children TB diagnosed	2 (2.6%)
Total	203		230		78

**Table 4:** Who informed index cases to bring children for TB screening?

Type of informant	N	%
Health care worker at OPD	5	10.4
Health care worker at TB clinic	29	60.4
Other health care worker in the facility	4	8.3%
Community health extension worker	5	10.4
Any person whom index case know	5	10.4%
Total	48	100

The 203 index cases together had 230 children age 0–5 years living in their household (Table 1), and the majority 140 (60.8%) were age 3 years and below. The 69 PTB index cases, who have reported to bring child contact for TB screening, all together they have brought 78 (33.9%) children for TB screening. For 76 (97.4%) children the screening result was negative and out of them 3 (3.9%) were offered IPT. Two children were diagnosed with PTB (Table 3).

In total, 60 HCWs were interviewed. Of them, 44(73.3%) were diploma clinical nurses and 16 (26.7%) were health officers. HCWs training history in the past 2 years indicated that, overall 28 (46.7%) were trained and by type of clinic, 15 (53.6%) in TB clinic, 9 (32%) in HIV clinic and 4 (14%) in the IMCI clinic were trained on TB and TB/HIV. When asked about their practice with regards to child contact screening, 50 (83%) of HCW indicated that they request index cases to bring their children for TB screening. However, when asked how many child contacts the HCW had screened in their clinic in the last quarter, 21 (35%) indicated they did not do contact screening and 5 (8.3%) indicated they referred all contacts to a higher level (hospital) for TB screening. Fourteen (23.3%) HCWs did not remember how many children they had screened as they do not keep records, 9 (15%) estimated the number screened from their memory, while 11 (18.3%) from the HIV clinic showed their record of screened in the quarter. Nearly all HCWs 59 (98.3%) believed families would bring their child contact for screening if requested. The most important challenge HCWs mentioned in screening children for TB was of difficulty to diagnose TB as children usually do not produce sputum 33(55%) and unavailability of chest X-ray 14(23%) (Table 5). Forty seven HCWs (78.3%) indicated they do not have any job aids for TB screening and IPT in children. Although 53 (88.3%) HCWs believed that IPT would benefit children to prevent TB, only 8 (13%) of interviewed HCWs reported to have ever put a child on IPT, while 52 (86.7%) said they have never prescribed IPT for children. When asking for the records on IPT provision, only 2 HCWs could show their records of children on IPT in the last quarter. A total of 22 (36.7%) HCWs said they do not record, while 20 (33%) said they do not provide IPT for children 0–5 years. And, perceived challenge of HCWs on IPT indicated that 28 (46.7%) said poor supply of child INH dose and 9(15%) fear of INH side effect in children (Table 6).

**Table 5:** HCWs perceived challenges on screening children.

What is the most important challenge in child TB screening?	N (%)
Difficulty to diagnose TB: (since children don’t produce sputum and AFB is usually negative)	33 (55)
Chest X-ray (CXR) is not available (low capacity)	14 (23)
Low awareness of child family (on contact screening)	5 (8.3)
There is no problem to screen a child contact	5 (8.3)
HCWs are not trained (skill/knowledge gap)	3 (5)
Total	60 (100)

**Table 6:** HCWs perceived challenges on IPT in children.

Most important challenge in IPT	N (%)
Parents wouldn’t bring their child for IPT	6 (10)
Supply problem of INH dose for children	28 (46.7)
Fear of INH side effect	9 (15)
Difficult to rule out TB in children	4 (6.7)
Mentioned more than one of the above problems	4 (6.7)
Did not see any problem	9 (15)
Total	60 (100)

## Discussion

In many instances in the health care system of different settings, it is common to miss opportunity in preventing TB among children. And the most important missed opportunities in childhood contact investigation were failure to detect source cases, failure to identify child contacts, delayed or incomplete evaluation of children exposed to TB, and inadequate treatment of latent TB infection [10].

This study examined the implementation of child contacts screening and provision of preventive treatment under routine program setting in Addis Ababa, Ethiopia. The national TB control program largely relies on passive child contacts screening by asking parents to bring their children to the health facility to be screened. Though we could not exclude a recall bias in this study, only a quarter of PTB index cases reported that they were requested by HCWs to bring their children for TB screening. We anticipate this low performance on child contact screening could even be worse in HFs with less patient volume and less work experience on DOTS. And, here we would like to emphasize that lack of documentation has been critical bottleneck in assessing performance of contact screening in children. In our finding nearly all (93.8%) index cases reported that they complied with the request of HCW, and this might be good news for the program suggesting that considerable proportion of household child contacts could be reached through passive contact investigation.

As per the national guidelines, all children under five who are at risk of active TB and where TB is ruled out, they should be offered IPT for six month. In our finding, only 3.9% (3/76) contacts received IPT. It seems there exist universal barrier in IPT provision in most high burden setting [11]. Different studies demonstrated variable degrees of but high missed opportunity for chemoprophylaxis in children. For instance, a study in Cape Town, South Africa showed that only 36.7% of eligible children were offered IPT [12], while another study in the same setting found only 1% of the eligible children received IPT [13], and in Malawi 6% of children got IPT [9]. It would be essential to note that in this study HCW have reported reasons for low IPT provision for child contacts screened negative were attributed to the challenges related to exclude TB in children, for instance the difficulty of sputum sample collection in young children, the low sensitivity of AFB test and poor access to CxR. From HCWs response we could implicitly understood that there is a skill and knowledge gap among HCWs in the approach for child contact screening as they have put more emphasis on access to CxR and the low sensitivity of AFB. Though the mentioned challenges have significant importance in the diagnosis of TB in young children, considerable proportion of child contacts could have been screened using simple clinical algorithm as primary tool in order to take appropriate measures such as IPT in those TB exposed children. TB program need to address this gap by improving HCWs competence through training, mentorship, job aids and supportive supervision. In addition, the TB control program need to track and ensure the availability of pediatric INH supply, as HCWs reported INH supply was one of the major challenges in IPT provision in children.

Emphasis on early effective interventions in children 1–5 years is for a good reason that, primary infection before 2 years of age frequently progressed to serious disease and in children < 3 years the presence of symptoms represent a window of opportunity in establishing diagnosis before progression to serious disease [14]. Important determinants of a child’s risk of developing TB after infection are age (children below 3 years of age) and immune status [14,15]. Two thirds (61%) of the reported contact children in this study were below 3 years of age. Though we could not determine the proportion of children infected of the reported total 230 exposed children, we have found that only 34% (78/230) of contacts were TB screened and 2 (2.6%) were diagnosed with TB. If TB screening were systematically done for all children, we estimate that twice as many TB cases could be identified. And, if we consider the risk of primary infection after exposure as 30% and risk of progression to TB disease as 10% in these age group of 1–5 years children, then 69 (6.9%) of TB exposed children had primary infection. Had it been not for the missed opportunity in preventing TB in these children by providing IPT, we could have averted 4.9 more future TB cases as 2 had already been TB diagnosed.

The key challenges perceived by HCWs in the implementation of child contact screening were difficulty to rule out TB disease due to often children do not produce sputum and chest x-ray (CxR) at primary health care level is usually not accessible. It’s probably due to the recommendation of TST and CxR as screening test to exclude TB in children that most HCWs consider CxR as mandatory test in child contact screening, while in fact it is not [6,7, 16]. Clear guidance and tools for HCWs especially at a primary health care level for a symptom-based screening approach as recommended by WHO is critical to address barrier related to child contact screening and preventive therapy [17].

Moreover, children do not necessarily access TB program for TB service rather often they are managed outside the TB program by child health workers. As this study indicated HCWs at the child health clinics are less trained than the HIV and TB clinic staff. Therefore, TB program need to train HCWs at the child health clinic in order to use them as a support to find and treat TB exposed or sick children [18].

It was observed that in this study documentation was poor and there was no format to keep track of child contact management in the program. In South Africa it was demonstrated that after the implementation of an IPT register, documented identification of child contacts, IPT initiation and adherence in TB exposed children has improved [19]. Similarly studies in India also shown improved contact screening and chemoprophylaxis and HCWs expressed satisfaction with the use of IPT card and register saying that it helped them to remember to complete required tasks [20,21]. Therefore, introduction of user friendly formats to monitor contact screening and management need to be considered and prioritized by the TB control program.

Family’s decision to bring their child contacts for TB screening to a health facility depends on multiple factors, for example, perceived susceptibility and awareness, access to care, etc [22, 23]. And, those index cases who failed to bring their household contacts were those not informed by HCW and had low awareness on child contact screening. Targeted messages and educational materials to improve family’s awareness on child contact screening are important.

The study was conducted in Addis Ababa town, where implementation of contact screening is assumed to be more feasible. As we have not investigated other areas of the country, we could not assume the findings of this study are representative of the whole nation. We could not reach the targeted sample size and it was mainly due to our assumption of proportion of households of index cases with children ≤ 5 yr of age was lower than anticipated and due to time and cost constraint that we could not continue until we reached the targeted sample size. Despite this we feel our study yielded valid results and answered our objectives.

## Conclusion

An important opportunity is missed to prevent and diagnose TB in children in Addis Ababa, Ethiopia as household child contact screening is not optimally implemented. Overall compliance of index cases for passive contact screening was found to be high, suggesting good proportion of child contacts could still be reached through passive contact screening, if the following programmatic gaps and challenges are addressed by the TB control program: 1) Capacity building of HCWs especially at primary health care level through trainings, mentorship, supportive supervision and availing clinical and screening algorithms. 2) Documentation of child contact screening would be an important source of information on performance and to generate local evidence 3) Ensuring the availability of pediatric INH supply is important 4) Improving public awareness is essential through various means of educational materials in order to augment demand for the service.

## Supporting Information

S1 File This is the file S1 Title. Patient interview questionnaire (1).
(DOCX)

S2 File This is the file S2 Title. Health care worker interview questionnaire (2).
(DOCX)
