# Supplementary material for: Cross Sectional Study Evaluating Routine Contact Investigation in Addis Ababa, Ethiopia: A Missed Opportunity to Prevent Tuberculosis in Children
Source: PLoS One. 2015 Jun 17;10(6):e0129135. doi: 10.1371/journal.pone.0129135 (PMC4470906; doi:10.1371/journal.pone.0129135)
Supplement: S1 File — Patient interview questionnaire (1). (DOCX) [file pone.0129135.s001.docx]

**Patient interview questionnaire (1)**

This form is **only** going to be filled when a smear positive PTB patient was on DOT for at least 02 weeks and has household contacts of children 0-5 year age.

Code number: _____________

**I) General Information** (information from TB unit register)

1. Health center: __________________

Sub-city: __________________

1. Date of interview: DD____ MM_____ YY__________
2. Age of patient (index): __________

1. Sex:
2. Female
3. Male
4. Type of TB (from TB register)
5. New smear positive PTB
6. Retreatment smear positive PTB
7. MDR-TB confirmed (smear /culture positive)
8. Treatment start date: DD____MM_____ YY_____
9. How long ago did the patients start anti-TB treatment?
10. 2 - 4 weeks
11. 5 - 8 weeks
12. 9 - 12 weeks
13. More than 12 weeks (Please specify _________________)

**II. Information on TB screening of children 0-5 years**

1. Level of education:
2. Illiterate
3. Elementary school
4. Secondary school
5. College (diploma or degree)
6. Monthly income:
7. Less than 500 birr / mth
8. From 500 – 1000 birr / mth
9. From 1000 – 2000 birr / mth
10. Above 2000 birr
11. I don’t have anything
12. Do the patient use any transport means to come to the HF for DOT?

i. Yes (please specify which _____________& amount of cost/day ___________)

ii. No I don’t use any

1. Did anyone request you to bring the child/children for TB screening?
2. Yes (go to 13)
3. No
4. Even if no one requested you, did you take your child to be screened for TB?
5. Yes I took him (go to 16)
6. No, I didn’t (go to 15)
7. Did you bring your child for TB screening, as requested?
8. Yes, I brought him
9. No, I didn’t
10. If someone requested you to bring the child /children, who did?
11. HCW at OPD
12. HCW at TB clinic
13. Other HCW in the health facility (specify _________________)
14. Community health worker: HEW
15. Heard it from media: e.g. radio / TV/ etc
16. Other (specify __________________)
17. If the child **was not brought for screening**, why didn’t you bring the child?
18. I don’t know whether he/she needs screening
19. I am not convinced that my child will benefit
20. I don’t think my child will get TB
21. My house is far
22. I don’t have money
23. I don’t have time
24. Other (specify________________________________________________________________________________________________________________________________)
25. Please fill the following table: how many children screened in the family, result, etc. If patient answered “I don’t know” for Q in column 4,5,6,7 please fill 99.

| 1 | 2 | 3 | 4 | 5 | 6 | 7 |
| --- | --- | --- | --- | --- | --- | --- |
|  | Age | Brought to HF for screening? (yes, no) | Screened: yes / no | Diagnosed TB: yes / no | Got IPT: yes/no | How long on IPT? |
| Child one |  |  |  |  |  |  |
| Child two |  |  |  |  |  |  |
| Child three |  |  |  |  |  |  |
| Child four |  |  |  |  |  |  |
