# Supplementary material for: Cross Sectional Study Evaluating Routine Contact Investigation in Addis Ababa, Ethiopia: A Missed Opportunity to Prevent Tuberculosis in Children
Source: PLoS One. 2015 Jun 17;10(6):e0129135. doi: 10.1371/journal.pone.0129135 (PMC4470906; doi:10.1371/journal.pone.0129135)
Supplement: S2 File — Health care worker interview questionnaire (2). (DOCX) [file pone.0129135.s002.docx]

**Health care worker interview questionnaire (2)**

**(HCW working in TB, HIV, MCH)**

Code number: _______

**I. General information:**

1. Health center: _________________

Sub-city: _________________

1. Interview date: DD ____MM_____YY___________
2. HCW qualification: ________________________
3. HCW responsibility & location: ___________________
4. Trained on TB, TB/HIV in the past 2 years:

i. Yes

ii. No

1. HCW age: __________ on Nov 2013
2. HCW Sex:

i. Female

ii. Male

**II. HCW practice information:**

1. How long have you been working in this clinic (TB, HIV or MCH)? _________
2. What do you do, if you know (find) a smear +ve PTB patient is living with children 0-5 years in the same household? **(NB. do not read choices for the interviewee)**
3. I don’t do anything
4. Let them to take to HF & screened for TB
5. Other (explain___________________________________)
6. Is there a recording and reporting format for TB contact screening? **(Observe!)**
7. Yes, there is
8. No, there is no
9. How many children 0-5 yrs have you screened for TB, in the last quarter?
10. Numbers of children screened /quarter______________(from record)
11. I don’t remember since I don’t have recording form
12. I don’t have a record, but it’s roughly around _________
13. We don’t do contact screening
14. Other ___________________________________
15. Do patients (i.e. PTB patients) accept / agree when you request them to bring children for TB screening?
16. Yes, they agree
17. No, they don’t agree (go to 14)
18. I’ve never requested them to bring (go to 14)
19. (If they agree with your advice), do they bring their child/children for TB screening?

i. Yes

ii. No

1. What do you think is the **most important challenge/s** for TB contact screening in children?
2. _____________________________
3. _____________________________
4. _____________________________
5. If patients do not bring children for screening (after your advice), what do you think is the reason?
6. They don’t think their children will get TB (or they will not be convinced)
7. They are reluctant and I don’t know why
8. They say that they don’t have money
9. Other reason, please specify __________________________
10. Do you have **job aid** on TB contact screening for children 0-5yrs?
11. Yes (please ask to observe)
12. No
13. When a family brings a child 0-5 yrs for TB screening, what is/are the procedures for TB screening? *(please don’t read the choices below for the respondent, let him/her answer & check all responses mentioned below)*
14. Ask for sign and symptom of TB
15. I will send for lab (AFB)
16. Send him for CXR
17. Refer the child, specify where _________________
18. I don’t know
19. Other (specify _________________________________________)
20. What do you do, if a child (0-5 yrs) screened **negative** (if child don’t have TB)?
21. Reassure the family & send back home
22. Will advise for IPT
23. Other (please specify _________________________________)
24. Do you think IPT will benefit a child (0-5 yrs) to protect him from TB?
25. Yes it has benefit
26. No, it doesn’t have benefit
27. I don’t know about it
28. Other, specify ______________________________
29. Will you please explain how INH (IPT) is prescribed for children 0-5 years (dose, duration)?
30. I don’t know
31. 100 mg daily for 6month
32. 10 mg /kg per day for 6 month
33. 300mg daily for 6 month
34. Other, specify ________________
35. Is there a job aid for IPT? **(Please, observe!!)**
36. Yes
37. No
38. Have you put any child 0-5 yrs on IPT after TB screening?
39. Yes
40. No, never
41. Is there a recording & reporting form for IPT (children)?
42. Yes (please observe)
43. No
44. How many children 0-5 yrs have you put on IPT in the past one quarter?
45. If recorded ________ per quarter
46. There is no record, I don’t know how many
47. No record, but roughly around ___________
48. We don’t provide IPT for children 0-5 yrs
49. Other ________________________________
50. What is the most important challenge/s for provision of IPT for children 0-5yrs? (please circle if more than one answer)
51. Parents or guardians don’t bring their children
52. There is no INH for children
53. We are afraid of side effects of INH
54. It’s very difficult to exclude TB disease in children
55. Other, please specify __________________________________________________________________________________________________________________________
